# The Impact of Patient Profiles and Procedures on Hospitalization Costs through Length of Stay in Community-Acquired Pneumonia Patients Based on a Japanese Administrative Database

**DOI:** 10.1371/journal.pone.0125284

**Published:** 2015-04-29

**Authors:** Hironori Uematsu, Susumu Kunisawa, Kazuto Yamashita, Yuichi Imanaka

**Affiliations:** 1 Department of Healthcare Economics and Quality Management, Graduate School of Medicine, Kyoto University, Kyoto City, Kyoto, Japan; 2 Department of Biomedical Sciences, Ritsumeikan University, Kyoto City, Kyoto, Japan; Azienda Ospedaliero-Universitaria Careggi, ITALY

## Abstract

**Background:**

Community-acquired pneumonia is a common cause of patient hospitalization, and its burden on health care systems is increasing in aging societies. In this study, we aimed to investigate the factors that affect hospitalization costs in community-acquired pneumonia patients while considering the intermediate influence of patient length of stay.

**Methods:**

Using a multi-institutional administrative claims database, we analyzed 30,041 patients hospitalized for community-acquired pneumonia who had been discharged between April 1, 2012 and September 30, 2013 from 289 acute care hospitals in Japan. Possible factors associated with hospitalization costs were investigated using structural equation modeling with length of stay as an intermediate variable. We calculated the direct, indirect (through length of stay), and total effects of the candidate factors on hospitalization costs in the model. Lastly, we calculated the ratio of indirect effects to direct effects for each factor.

**Results:**

The structural equation model showed that higher disease severities (using A-DROP, Barthel Index, and Charlson Comorbidity Index scores), use of mechanical ventilation, and tube feeding were associated with higher hospitalization costs, regardless of the intermediate influence of length of stay. The severity factors were also associated with longer length of stay durations. The ratio of indirect effects to direct effects on total hospitalization costs showed that the former was greater than the latter in the factors, except in the use of mechanical ventilation.

**Conclusions:**

Our structural equation modeling analysis indicated that patient profiles and procedures impacted on hospitalization costs both directly and indirectly. Furthermore, the profiles were generally shown to have greater indirect effects (through length of stay) on hospitalization costs than direct effects. These findings may be useful in supporting the more appropriate distribution of health care resources.

## Introduction

Community-acquired pneumonia (CAP) is a common infectious disease and a major cause of hospitalization that places a heavy burden on health care systems.[1] In Japan, the Ministry of Health, Labour and Welfare (MHLW) estimated that there were 38,000 hospitalizations per month due to pneumonia in 2011, which was twice the number of cases in 1996.[2] The economic burden of CAP on health care systems is expected to increase in aging societies due to the higher susceptibility of elderly persons to pneumonia and pneumonia-related complications.[3] Thus, it is becoming increasingly important to improve and optimize the use of health care resources in CAP treatment, especially in aging populations such as Japan.

In 2003, the Japanese government began the implementation of the Diagnostic Procedure Combination/Per-Diem Payment System (DPC/PDPS) for reimbursements to acute care hospitals under the public medical insurance scheme. The DPC/PDPS is similar to the US prospective payment system with diagnosis-related groups (DRG/PPS), and was implemented with the aim of reducing hospitalizations and length of stay (LOS) without decreasing quality of care.[4,5] However, the current DPC/PDPS has not been able to achieve appropriate reimbursements for medical resource use in CAP patients due to an inadequate consideration of patient severity and comorbidities, despite the wide variations in CAP inpatient severity in Japan.[6]

The Japanese MHLW is considering the integration of a “Comorbidity Complication Procedure” (CCP) matrix into the current DPC/PDPS in the near future. The CCP matrix would enable analysts to account for variations in patient severity and comorbidities when investigating the use of health care resources. In order to develop an appropriate CCP matrix for the DPC/PDPS, it is first necessary to ascertain the degree of influence of patient severity, comorbidity, and procedures on medical expenses in each disease category. Several studies have previously reported the hospitalization expenses required to treat CAP.[7,8] However, there are few existing studies that have investigated the costs to treat CAP patients stratified according to their risk profiles and procedures.[9]

LOS is a major factor to consider when examining the relationship between patient severity and hospitalization costs because there is a high possibility that LOS acts both as an intermediate variable and an explanatory variable for costs. A previous study has reported that the LOS in CAP inpatients has a high degree of influence on hospital costs.[10] In addition, other studies have reported that pneumonia severity, comorbidities, and specific procedures (such as the use of mechanical ventilation) are associated with prolonged LOS in CAP patients.[11–13] These studies indicate that LOS is a possible intermediate variable between patient severity and costs.

A structural equation modeling (SEM) method may be able to clarify the relationship described above. SEMs are applications of traditional multivariate analyses, and a response variable in one regression equation in an SEM may become a predictor in another equation. Variables in an SEM can demonstrate relationships with one another either directly or through other variables as intermediaries.[14]

The purpose of our study was to investigate how patient risk profiles and procedures affect hospitalization costs in CAP patients while taking into account LOS variations through the use of an SEM in order to contribute to the establishment of a more appropriate payment system in the future.

## Methods

### Data source

We obtained patient data from the Quality Indicator/Improvement Project (QIP), which regularly collects administrative claims data from voluntary participant acute care hospitals in Japan. In 2014, there were 388 QIP participant hospitals, which varied in scale, region, and health care provider type.

All participant hospitals provide data to the QIP in the Japanese DPC data format, which is a case mix classification system for reimbursements under the public medical insurance scheme.[15] The DPC data include clinical/discharge summaries and administrative claims information. Hospital identifiers, patient demographics, admission and discharge statuses, major diagnoses, and comorbidities are included in the clinical summary data. A modified version of the Barthel Index (BI), which consists of 10 items that measure a person’s performance in their activities of daily living (ADL), is included as a cumulative score ranging from 0 to 20 points.[16] Diseases were identified through International Classification of Diseases, 10^th^ Revision (ICD-10) codes. Clinical information about the A-DROP pneumonia severity scoring system, which is a modified version of CURB-65, is also included in these data.[15] The type, number, date, and cost of each clinical procedure performed are included in the administrative claims information component of the data.

### Study inclusion and exclusion criteria

**Fig 1:**
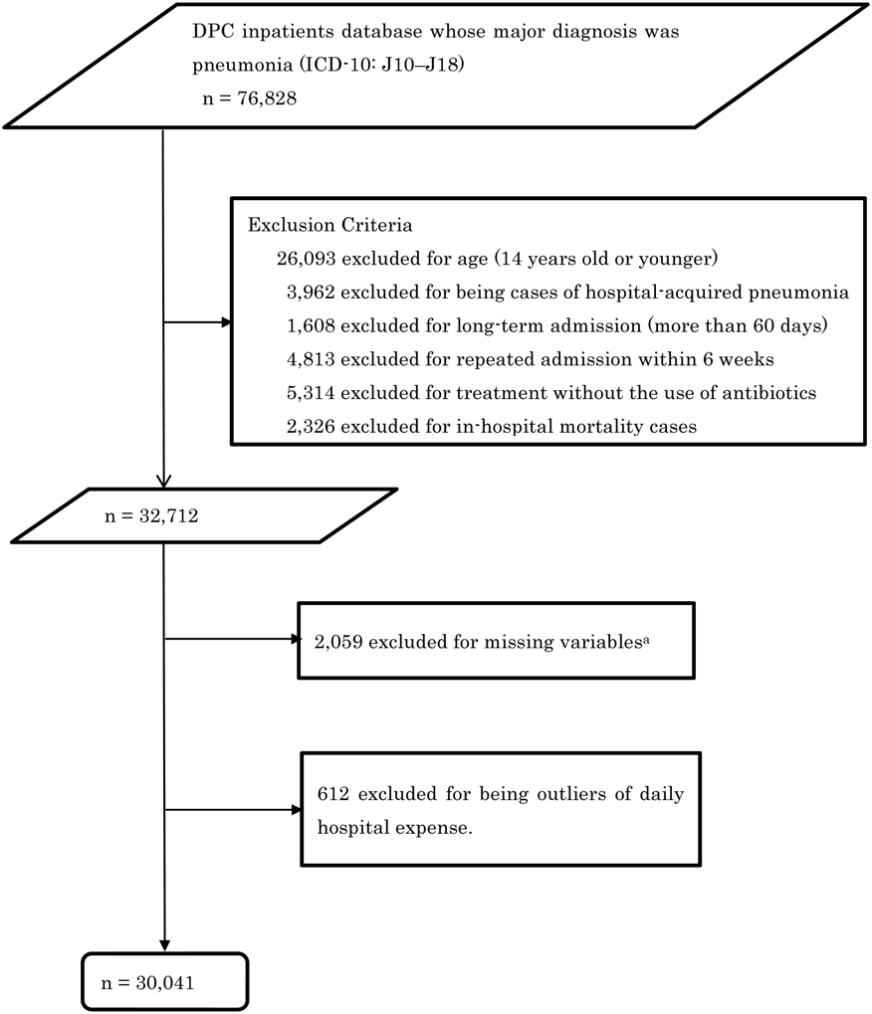
Selection of inpatients with community-acquired pneumonia from the Diagnostic Procedure Combination (DPC) database. ^a^Missing variables included blood urea nitrogen or dehydration, respiratory state, orientation, blood pressure, number of beds, number of physician, and number of nurses

Fig 1 shows the patient selection process. We selected inpatients who had been discharged from the study hospitals between April 1, 2012 and September 30, 2013, and whose major diagnosis was pneumonia (ICD-10: J10–J18). Patients were excluded if they were aged 14 years or younger, had hospital-acquired pneumonia, were hospitalized as a readmission within 6 weeks of being discharged, had been hospitalized for more than 60 days (long-term hospitalization), had not been administered an antibiotic within 2 days of admission, or had died during hospitalization. Patients were also excluded as outliers if their daily hospitalization costs were in the top or bottom 1 percentile.

### Hospitalization costs calculation

Total hospitalization costs were calculated as the aggregate of all fees for health care services incurred during hospitalization.[17] Services include basic and special inpatient care, initial consultation and examination, imaging, pharmacy, injections, treatments, invasive procedures, and pre-discharge guidance; fees were calculated in Japanese yen. All-cause costs (i.e., costs not directly related to CAP) during hospitalization were also included in the total hospitalization costs, because CAP can result in the deterioration of other diseases. Costs were converted from Japanese yen to US dollars (US$1 = \104) based on purchasing power parities in 2013.

### Statistical analysis

The primary endpoint for the analysis was total hospitalization costs. Baseline patient and hospital characteristics were first presented as descriptive statistics, including means, standard deviations, medians, interquartile ranges, minimum values, and maximum values. We drew box plots to illustrate the unadjusted relationships between patient states and total hospitalization costs. In addition, Spearman correlation coefficients were calculated to provide insight into the unadjusted relationships of each variable (S1 Table).

**Fig 2:**
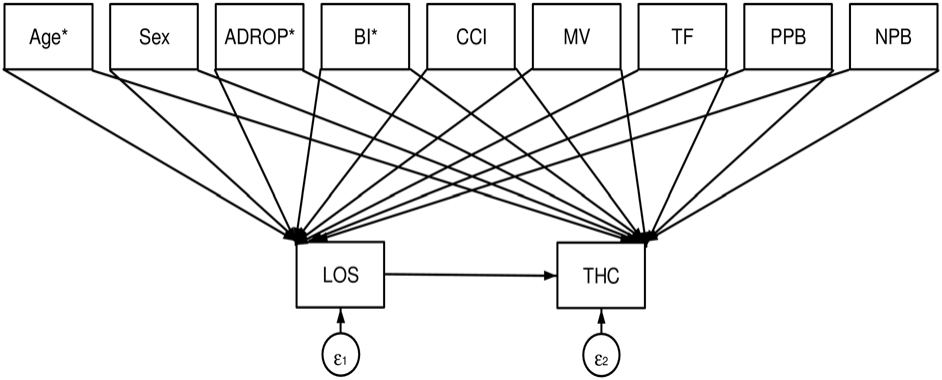
A path showing the relationship between each variable using structural equation modeling. Abbreviations: BI, Barthel index; CCI, Charlson comorbidity index; MV, Mechanical ventilator; TF, Tube feeding; PPB, Physicians per beds; NPB, Nurses per beds; LOS, Length of stay; THC, Total hospitalization costs; ε; error term *Age, ADROP scores, Barthel index and Charlson comorbidity index include dummy variables in each box.

SEM analysis was conducted using LOS as an intermediate variable. Fig 2 shows the path diagram of the model, which presents the relationship between each variable. From the DPC data, we explored the following candidate independent variables identified from existing evidence [10–13] or based on clnical rationare: patient age, sex, A-DROP score, BI score, Charlson Comorbidity Index (CCI) score (Dartmouth-Manitoba version [18]), mechanical ventilation use, tube feeding (enteral nutrition), and LOS. In order to adjust for differences in hospital structural characteristics, we also included the number of physicians per 10 beds and number of nurses per bed as independent variables. Patient age, A-DROP scores, BI scores, and CCI scores were measured upon admission. These variables were included in analysis as ordinal dummy variables, and their cutoff points were determined using the interquartile range. All variables were analyzed as observation variables in this model.

We defined 3 path effects for the SEM: “Direct effects”, referring to the effects of the candidate factors on hospitalization costs that bypass LOS; “Indirect effects”, referring to the effects of the candidate factors on hospitalization costs through LOS; and “Total effects”, referring to the sum of the direct and indirect effects. Lastly, we calculated the ratio of indirect effects to direct effects for each factor, and compared which effects had a greater impact on total hospitalization costs using a chi-square test.

All statistical analyses were performed using SPSS software, version 20 (SPSS Inc., Chicago, IL, USA) and STATA 13 statistical software (STATA Corp, College Station, TX, USA) for Windows. Statistical significance was set at P < 0.05.

### Ethical standard

The collection and analysis of DPC data from the QIP hospitals were approved (Approval Number: E-05) by the Ethics Committee of Kyoto University Graduate School of Medicine. This study was conducted in accordance with the Ethical Guidelines for Epidemiological Research established by the Japanese national government, which stipulates the requirements for protecting patient anonymity. Based on these guidelines, our study satisfied the necessary conditions to waive the need for informed consent, and the ethics committee approved that waiving.

## Results

### Patient characteristics

We analyzed 30,041 CAP patients from 289 hospitals. Patient and hospital characteristics are shown in Table 1. The mean and median patient ages were 72.8 years and 78.0 years, respectively; men comprised 58% of the study sample. The mean A-DROP score, which has a maximum value of 5, was 1.49. The number of hospital beds ranged from 30 to 1,151 among the hospitals. There was approximately one physician for every 5 beds in the sample. The mean, median, and standard deviation of LOS were 13.9 days, 11.0 days and 9.56 days, respectively. The mean, median and standard deviation of total hospitalization costs were US$4,781, US$2,971, and US$409, respectively.

**Table 1:** Characteristics of 30,041 pneumonia patients and 289 hospitals.

Patient characteristics	Mean	SD	Median	IQR	Min	Max
Age, years	72.8	18.2	78.0	66–85	15	107
Sex (0: Female, 1: Male)	0.58	0.49	1	0–1	0	1
A-DROP score^a^	1.49	1.12	1	1–2	0	5
Barthel Index	13.5	7.79	19	7–20	0	20
Charlson Comorbidity Index	1.06	1.13	1	0–2	0	8
Mechanical ventilation	0.018	0.11	0	0–0	0	1
Tube feeding	0.025	0.16	0	0–0	0	1
Length of stay, days	13.9	9.56	11.0	8–17	1	60
Total hospitalization costs, US$	4781	2971	4009	3006–5965	366.7	36253
Hospital characteristics						
Number of beds	329	188	295	192–437	30	1151
Number of physicians	66	59.3	48.0	25–87	3	432
Number of nurses	264	178	213	132–355	26	1210
Number of physicians per 10 beds	1.80	0.67	1.68	1.35–2.17	0.31	4.35
Number of nurses per bed	0.79	0.19	0.77	0.66–0.91	0.27	1.47

Abbreviations: SD, standard deviation; IQR, interquartile range

^a^A-DROP score: Pneumonia severity scoring system that is a modified version of CURB-65

**Fig 3:**
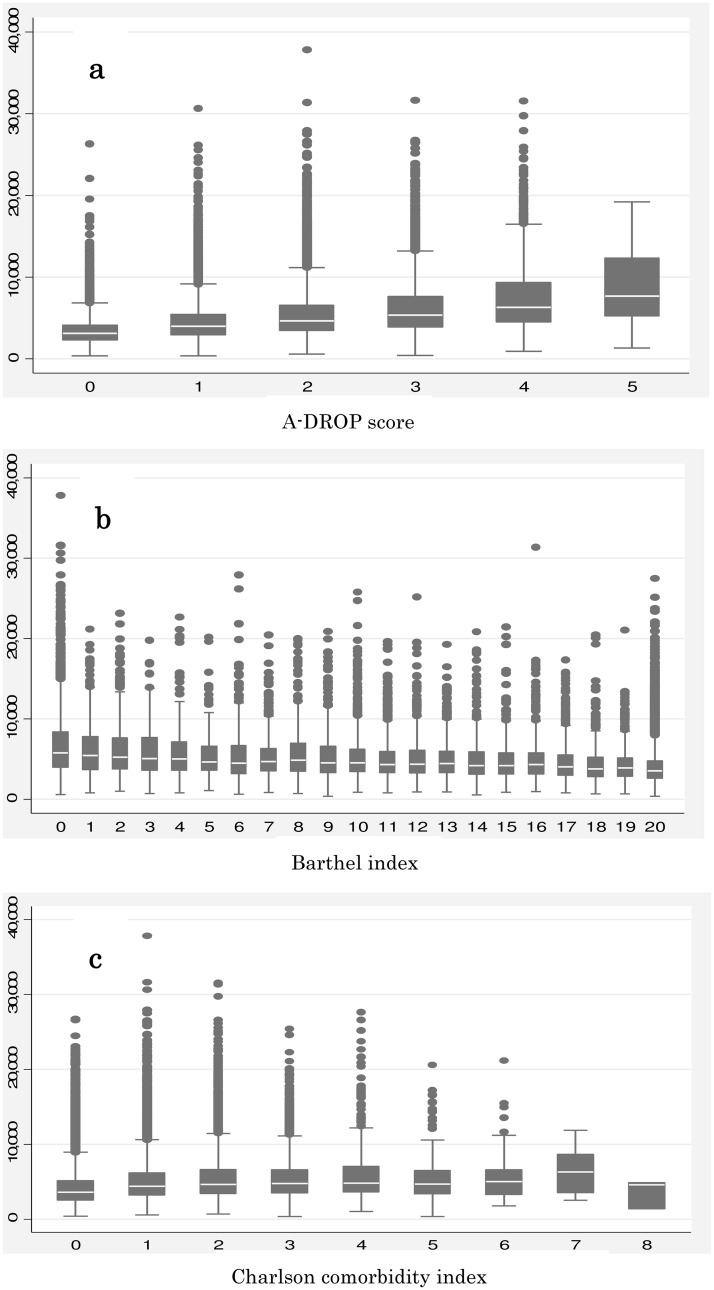
Box plots of hospital costs by patient severitys. (a) Box plot of hospital costs by A-DROP score (b) Box plot of hospital costs by Barthel index (c) Box plot of hospital costs by Charlson comorbidity index. All horizontal axes indicate total hospitalization costs(US$) and the vertical axes indicate (a) A-DROP score (b)Barthel index (c)Charlson comorbidity index.

Fig 3 shows the unadjusted relationships between hospitalization costs and patient severity (A-DROP score, BI, and CCI) using box plots. The results showed that higher severity was related with higher hospitalization costs, except in CCI scores of 5 or more.

### Structural equation modeling

Table 2 shows the impact of the candidate variables on LOS in an SEM analysis. All candidate variables, except patient sex and the number of medical staff per bed, had a significant and positive association with increased LOS. Ordinal dummy variables such as age, A-DROP scores, BI scores, and CCI scores showed dose-response relationships with LOS. The use of mechanical ventilation and tube feeding were associated with LOS extensions of 7.79 days and 8.34 days, respectively.

**Table 2:** Direct effects of the factors on length of stay using a structural equation model (n = 30,041).

Variables	B	95% CI Lower	95% CI Upper	P-value
Sex (Male)	0.10	-0.11	0.30	0.36
Age (Reference: 15–64 years)	Reference			
65–74 years	1.77	1.43	2.11	<0.001
75–84 years	1.97	1.60	2.33	<0.001
≥85 years	2.78	2.39	3.16	<0.001
A-DROP score (Reference: Mild, 0)	Reference			
Moderate 1–2	1.53	1.19	1.88	<0.001
Severe 3–5	3.81	3.38	4.25	<0.001
Barthel Index (Reference: Good, 20)	Reference			
Fair 8–19	1.03	0.76	1.30	<0.001
Poor 0–7	3.57	3.26	3.87	<0.001
Missing Barthel Index data^a^	2.05	1.72	2.38	<0.001
Charlson Comorbidity Index (Reference: None, 0)	Reference			
Moderate 1	1.42	1.18	1.66	<0.001
High ≥2	2.01	1.76	2.27	<0.001
Mechanical ventilation	7.79	7.03	8.55	<0.001
Tube feeding	8.34	7.68	8.99	<0.001
Number of physicians per 10 beds	-0.60	-0.77	-0.44	<0.001
Number of nurses per bed	-0.92	-1.52	-0.32	0.003

Abbreviations: B, Unstandardized coefficient; CI, confidence interval

^a^Missing Barthel Index data: Barthel Index data were missing in 4,100 of the 30,041 pneumonia patients

Table 3 presents the results of the SEM analysis for total hospitalization costs based on the path diagram shown in Fig 2. For direct effects, the results showed that except for patient age, the candidate variables were positively associated with increased total hospitalization costs. For indirect effects, the results showed that except for the number of medical staff per bed, the candidate variables were positively associated with increased total hospitalization costs. A-DROP scores, BI scores, and CCI scores showed dose-response relationships with hospitalization costs in both direct and indirect effects. Mechanical ventilation use was associated with increased hospitalization costs, both directly (unstandardized coefficient: 2,629; 95% CI: 2,536–2,722) and indirectly (unstandardized coefficient: 2,128; 95% CI: 1,921–2,335). In tube feeding, the indirect effects (unstandardized coefficient: 2,277; 95% CI: 2,097–2,456) were found to be greater than the direct effects (unstandardized coefficient: 615; 95% CI: 534–695). In total effects, which are the sum of the direct and indirect effects, all candidate variables were statistically associated with increased total hospitalization costs, with the exception of the number of nurses per bed.

**Table 3:** Direct, indirect, and total effects of the factors on total hospitalization costs using a structural equation model (n = 30,041).

Variables	Direct Effects			Indirect Effects			Total Effects		
	→ THC			→ LOS → THC			→ THC + (→ LOS → THC)		
	B	95% CI		B	95% CI		B	95% CI	
Length of stay, days	273	272	274	No path			273	272	274
Sex (Male)	80	55	105	26	-30	83	106	45	168
Age (Reference: 15–64 years)	Reference								
65–74 years	-37	-78	4	484	391	577	447	346	549
75–84 years	-196	-240	-152	537	437	636	341	232	450
≥85 years	-301	-348	-254	758	653	864	457	342	572
A-DROP score (Reference: Mild, 0)	Reference								
Moderate 1–2	233	191	275	418	324	512	651	548	754
Severe 3–5	495	441	548	1041	922	1161	1536	1405	1666
Barthel Index (Reference: Good, 20)	Reference								
Fair 8–19	133	101	166	282	208	355	415	334	496
Poor 0–7	191	154	228	974	891	1057	1165	1075	1255
Missing Barthel Index data^a^	245	205	285	560	471	649	805	707	903
Charlson Comorbidity Index (Reference: None, 0)	Reference								
Moderate 1	108	79	137	388	323	453	496	425	568
High ≥2	191	159	222	550	323	453	740	664	816
Mechanical ventilation	2629	2536	2722	2128	1921	2335	4757	4530	4984
Tube feeding	615	534	695	2277	2097	2456	2892	2695	3088
Number of physicians per 10 beds	351	330	371	-165	-211	-119	186	136	236
Number of nurses per bed	343	269	416	-251	-415	-86	92	-88	272

Abbreviations: THC, Total hospitalization costs; LOS, Length of stay; B, Unstandardized coefficient; CI, confidence interval; AIC, Akaike information criterion; CFI, Comparative fit index; RMSEA, Root mean square error of approximation

AIC = 997634 CFI = 1.000 RMSEA = 0.000

^a^Barthel Index data missing: Barthel Index data were missing in 4,100 of the 30,041 pneumonia patients

Table 4 shows the ratios of indirect effects to direct effects for each candidate variable. The indirect effects were significantly stronger than the direct effects in A-DROP scores (severe A-DROP: 2.11; 95% CI: 1.77–2.44), BI scores (poor BI: 5.10; 95% CI: 4.02–6.18), CCI scores (high CCI: 2.88; 95% CI: 2.29–3.6), and tube feeding (tube feeding: 3.70; 95% CI: 3.13–4.27). The ratios of the age variable showed negative values because their direct effects were negative, despite the indirect effects being positive.

**Table 4:** Ratios of the indirect effects to the direct effects on total hospitalization costs (n = 30,041).

Variables	Ratio^a^	95% CI Lower	95% CI Upper	P-value
Sex (Male)	0.33	-0.38	1.04	0.064
Age (Reference: 15–64 years)				
65–74 years	-13.12	-28.03	1.80	0.064
75–84 years	-2.74	-3.54	-1.94	<0.001
≥85 years	-2.52	-3.04	-1.99	<0.001
A-DROP score (Reference: Mild, 0)				
Moderate 1–2	1.80	1.28	2.32	0.003
Severe 3–5	2.11	1.77	2.44	<0.001
Barthel Index (Reference: Good, 20)				
Fair 8–19	2.11	1.35	2.87	<0.001
Poor 0–7	5.10	4.02	6.18	<0.001
Missing Barthel Index data^b^	2.28	1.76	2.80	<0.001
Charlson Comorbidity Index (Reference: None, 0)				
Moderate 1	3.60	2.45	4.74	<0.001
High ≥2	2.88	2.29	3.48	<0.001
Mechanical ventilation	0.81	0.73	0.89	<0.001
Tube feeding	3.70	3.13	4.27	<0.001
Number of physicians per 10 beds	-0.47	-0.60	-0.34	<0.001
Number of nurses per bed	-0.73	-1.24	-0.23	<0.001

Abbreviations: CI, confidence interval

^a^Ratio; Effect ratios were derived from dividing indirect effects by direct effects

^b^Missing Barthel Index data: Barthel Index data were missing in 4,100 of the 30,041 pneumonia patients

## Discussion

In this study, we explored the factors associated with increased hospitalization costs in CAP patients using Japanese administrative data. Using 30,041 patients from 289 hospitals, SEM analysis demonstrated that A-DROP scores, BI scores, CCI scores, mechanical ventilation use, and tube feeding were statistically associated with increased hospitalization costs, regardless of whether LOS was included as an intermediate variable. Furthermore, the candidate variables (except for mechanical ventilation use) were more strongly associated with increased hospitalization costs through the LOS path.

At present, there are no multi-center studies that have evaluated the hospitalization costs to treat CAP in adult patients using validated patient risk profile scores such as A-DROP scores, BI scores, and CCI scores. To the best of our knowledge, there has only been one published report that evaluated CAP costs by patient profiles: Sato et al. [9] reported that CAP patients with high-risk profiles tend to increase treatment costs. Although that study analyzed CAP in adults in the US according to patient risk profiles, only age and comorbidities were included in the profiles. Our study is the first to assess hospitalization costs in adult CAP patients using a detailed patient profile that includes a pneumonia severity score and a patient ADL index.

Our study is also the first to report a dose-response relationship between the BI and LOS, although several studies have previously explored LOS prediction factors in pneumonia patients.[11–13] The BI was originally developed as a reliable measure of physical disability, mainly for patients with cerebrovascular diseases. However, Olga et al. [19] reported that lower BI scores were a predictive factor for short- and long-term mortality in elderly CAP patients. Their study indicated that poorer performance in the BI is a factor of pneumonia severity in pneumonia patients, which corroborates our finding of the significant association between higher BI scores and increased LOS.

Our results also showed that the direct effects of age had a negative dose-response relationship with hospitalization costs, although age has previously been shown to be a factor of severity.[20] With reference to existing reports, we postulate 2 possible explanations for this finding: the first is that elderly people tend to have lower utilization of intensive care because they may place a greater emphasis on the quality of life.[21] The other possible reason is that elderly people in Japan tend to remain in hospitals even if they do not require intensive care, which is a phenomenon termed “social hospitalization”.[22] Further investigation as to why increased age was associated with decreased costs after adjusting for LOS are needed to shed further light on this issue.

Our study suggests that the most severe patient profiles or procedures may impact on hospitalization costs through the prolongation of LOS. The candidate variables (except the use of mechanical ventilation and the number of medical staff per bed) were associated with total hospitalization costs more strongly through a LOS path (indirect effects path) than when bypassing the LOS path (direct effects path). Previous reports have noted the substantial influence of LOS on health care costs in CAP patients.[10,23] Before we conducted the SEM analysis, we had performed preliminary univariate analyses of each candidate variable (data not shown). The coefficient of determination of the preliminary simple model for LOS and hospitalization costs was 0.81, indicating that most of the variations in costs could be explained by LOS alone. In addition, Table 2 showed that with the exception of the number of medical staff per bed, each variable was statistically associated with increased LOS. Therefore, our study indicates that LOS is an essential intermediate variable for analyses of the relationship between patient profiles and hospitalization costs.

The impact of LOS as an intermediate variable on hospitalization costs varied greatly among the candidate factors. In Table 4, the ratios of indirect effects to direct effects indicate the impact of LOS on hospitalization costs. Although poor BI performance and tube feeding had high ratios, the use of mechanical ventilation had a ratio below 1.0. This suggests that the first 2 variables required a long period to treat, with a relatively small amount of medical supplies used per day. In contrast, higher numbers of medical staff resulted in shorter periods of treatment, but required the use of a larger amount of medical supplies per day. The varying importance of LOS impact on hospitalization costs should therefore be considered for the different patient profiles and procedures in future analyses.

### Limitations

This study has several potential limitations. First, our sample may not necessarily be representative of all CAP cases in Japan, as we did not include patients whose major diagnosis was either respiratory failure or sepsis with a secondary diagnosis of pneumonia. Our study sample had a relatively low average A-DROP score (1.49 points), which may have been due to the exclusion of these severe patients. The non-inclusion of these patients could therefore potentially confound our results.[24]

Second, there are only 4 coding slots for comorbidities in the Japanese DPC database. Therefore, the reported comorbidities in this study may be lower than the actual incidences. However, a previous study that reported the distribution of CCI for pneumonia in Denmark suggests that the CCI range of our sample was not inordinately low.[25] In any case, the coding in the DPC system is expected to improve in the near future, allowing the inclusion of more comorbidities.

Third, total hospitalization costs in this study were calculated by the sum of all fees of each medical practice, the prices of which are unique and determined by the advisory committee of the MHLW in Japan. This may reduce the direct generalizability of our results to other countries due to differences in fee schedules. However, these findings provide insight into the possible applications of disease severity information in reimbursement systems for both research and reform purposes, irrespective of national or regional systems.

Finally, there is an advanced analytical method in which hospitalization costs (as a response variable) are analyzed with a gamma distribution and a log link function using a regression model.[26] In our study, however, we used a normal distribution with an identify link instead of a gamma distribution, as it allowed us to calculate unstandardized coefficients. For confirmation purposes, we conducted additional generalized SEM analyses using gamma and Poisson distributions (S2 Table), which corroborated our results.

## Conclusions

In this study of 30,041 CAP patients in Japan, we analyzed the patient profiles, procedures, and hospital characteristics that impact on hospitalization costs using Japanese administrative claims data incorporated with clinical data. Our results showed that pneumonia severity, physical disability, comorbidities, use of mechanical ventilation, and tube feeding had both direct and indirect effects on hospitalization costs. Furthermore, it showed that patient profiles had stronger indirect effects on hospitalization costs through LOS than direct effects. Our findings may have applications in supporting the more appropriate distribution of health care resources in the future.

## Supporting Information

S1 Table Spearman correlation coefficients for total hospitalization costs, length of stay, patient profile/procedures and hospital characteristics (n = 30,041). All pairwise correlation coefficients were calculated using all available data. * Correlation is significant at the 0.05 level. ** Correlation is significant at the 0.01 level. Abbreviations: THC, Total hospitalization costs; LOS, Length of stay; BI, Barthel index; CCI, Charlson comorbidity index; MV, Mechanical ventilator; TF, Tube feeding; PPB, Physicians per bed; NPB, Nurses per bed. BI^a^: Barthel Index data were missing in 4,100 of the 30,041 pneumonia patients.
(PDF)

S2 Table Direct effects of the variables on total hospitalization costs and length of stay using a generalized structural equation model (n = 30,041). Abbreviations: CI, confidence interval. ^a^Hospitalization costs were analyzed using a gamma distribution with a log link. ^b^Length of stay was analyzed using a Poisson distribution with a log link. Log likelihood = -381619.15.
(PDF)
