# Supplementary material for: The Impact of Patient Profiles and Procedures on Hospitalization Costs through Length of Stay in Community-Acquired Pneumonia Patients Based on a Japanese Administrative Database
Source: PLoS One. 2015 Apr 29;10(4):e0125284. doi: 10.1371/journal.pone.0125284 (PMC4414582; doi:10.1371/journal.pone.0125284)
Supplement: S1 Table — All pairwise correlation coefficients were calculated using all available data. * Correlation is significant at the 0.05 level. ** Correlation is significant at the 0.01 level. Abbreviations: THC, Total hospitalization costs; LOS, Length of stay; BI, Barthel index; CCI, Charlson comorbidity index; MV, Mechanical ventilator; TF, Tube feeding; PPB, Physicians per bed; NPB, Nurses per bed. BIa: Barthel Index data were missing in 4,100 of the 30,041 pneumonia patients. (PDF) [file pone.0125284.s001.pdf]

S1 Table.

Spearman correlation coefficients for total hospitalization costs, length of stay, patient profile/procedures and hospital characteristics (n = 30,041)

Variable	THC	LOS	Sex	Age	A-DROP	BI	CCI	MV	TF	PPB	NPB
THC	1.00										
LOS	0.93**	1.00									
Sex	0.04**	0.02**	1.00								
Age	0.28**	0.30**	-0.08**	1.00							
A-DROP	0.38**	0.34**	0.07**	0.62**	1.00						
BI[a]	-0.33**	-0.30**	0.10**	-0.47**	-0.49**	1.00					
CCI	0.21**	0.18**	0.13**	0.15**	0.19**	-0.08**	1.00				
MV	0.17**	0.13**	0.01*	-0.01*	0.09**	-0.09**	0.04**	1.00			
TF	0.16**	0.16**	-0.01	0.03**	0.11**	-0.22**	-0.01*	0.17**	1.00		
PPB	0.05**	-0.06**	0.03**	-0.06**	-0.01	0.05**	0.02**	0.04**	-0.01	1.00	
NPB	0.04**	-0.03**	0.01*	-0.02**	0.02**	0.00	0.05**	0.03**	0.01	0.47**	1.00

All pairwise correlation coefficients were calculated using all available data

* Correlation is significant at the 0.05 level

** Correlation is significant at the 0.01 level

Abbreviations: THC, Total hospitalization costs; LOS, Length of stay; BI, Barthel index; CCI, Charlson comorbidity index; MV, Mechanical ventilator; TF, Tube feeding; PPB, Physicians per bed; NPB, Nurses per bed

BI[a] : Barthel Index data were missing in 4,100 of the 30,041 pneumonia patients
