# Supplementary material for: The Impact of Patient Profiles and Procedures on Hospitalization Costs through Length of Stay in Community-Acquired Pneumonia Patients Based on a Japanese Administrative Database
Source: PLoS One. 2015 Apr 29;10(4):e0125284. doi: 10.1371/journal.pone.0125284 (PMC4414582; doi:10.1371/journal.pone.0125284)
Supplement: S2 Table — Abbreviations: CI, confidence interval. aHospitalization costs were analyzed using a gamma distribution with a log link. bLength of stay was analyzed using a Poisson distribution with a log link. Log likelihood = -381619.15. (PDF) [file pone.0125284.s002.pdf]

S2 Table. Direct effects of the variables on total hospitalization costs and length of stay using a generalized structural equation model (n = 30,041).

Variables	Odds Ratio	Standard Error	95% CI Lower	95% CI Upper	P-value
Total hospitalization costs[a] ←					
Sex	1.02	0.003	1.01	1.02	<0.001
Age (Reference: 15-64 years)			Reference		
65-74 years	1.04	0.005	1.03	1.05	<0.001
75-84 years	1.00	0.005	0.99	1.01	0.573
≥85 years	0.98	0.005	0.97	0.99	<0.001
A-DROP scores (Reference: Mild, 0)			Reference		
Moderate 1-2	1.09	0.005	1.08	1.10	<0.001
Severe 3-5	1.15	0.007	1.13	1.16	<0.001
Barthel Index (Reference: Good, 20)			Reference		
Fair 8-19	1.04	0.004	1.03	1.05	<0.001
Poor 0-7	1.06	0.005	1.05	1.07	<0.001
Missing Barthel Index data[b]	1.07	0.005	1.06	1.08	<0.001
Charlson Comorbidity Index (Reference: None, 0)			Reference		
Moderate 1	1.05	0.004	1.04	1.05	<0.001
High ≥2	1.07	0.004	1.06	1.08	<0.001
Mechanical ventilation	1.30	0.014	1.27	1.32	<0.001
Tube feeding	1.02	0.010	1.00	1.04	0.064
Number of physicians per 10 beds	1.08	0.003	1.07	1.08	<0.001
Number of nurses per bed	1.05	0.009	1.03	1.06	<0.001
Length of stay, days	1.05	0.0002	1.05	1.05	<0.001
Length of stay[b] ←					
Sex	1.01	0.003	1.00	1.01	0.030
Age (Reference: 15-64 years)			Reference		
65-74 years	1.17	0.007	1.16	1.19	<0.001
75-84 years	1.18	0.007	1.17	1.19	<0.001
≥85 years	1.24	0.008	1.23	1.26	<0.001
A-DROP scores (Reference: Mild, 0)			Reference		
Moderate 1-2	1.17	0.007	1.16	1.19	<0.001
Severe 3-5	1.34	0.009	1.32	1.36	<0.001
Barthel Index (Reference: Good, 20)			Reference		
Fair 8-19	1.09	0.005	1.08	1.10	<0.001
Poor 0-7	1.28	0.006	1.27	1.30	<0.001
Missing Barthel Index data	1.18	0.006	1.16	1.19	<0.001
Charlson Comorbidity Index (Reference: None, 0)			Reference		
Moderate 1	1.11	0.004	1.10	1.12	<0.001
High ≥2	1.16	0.005	1.15	1.17	<0.001

Mechanical ventilation	1.48	0.013	1.45	1.50	<0.001
Tube feeding	1.49	0.012	1.47	1.51	<0.001
Number of physicians per 10 beds	0.96	0.003	0.95	0.96	<0.001
Number of nurses per bed	0.94	0.009	0.92	0.95	<0.001

Log likelihood = -381619.15

Abbreviations: CI, confidence interval

[a] Hospitalization costs was analyzed using a gamma distribution with a log link.

[b] Length of stay was analyzed using a Poisson distribution with a log link.
